# Subjective Evaluation of Female Adult Body Fat Distribution: A Scoping Review

**DOI:** 10.1111/obr.70068

**Published:** 2025-12-16

**Authors:** Susan C. Lennie, Andy Hall, Giang Nguyen, Anna Boath, Luke Vale, M. Dawn Teare, Nicola Heslehurst

**Affiliations:** ^1^ Population Health Sciences Institute Newcastle University Newcastle upon Tyne UK; ^2^ School of Pharmacy, Applied Sciences and Public Health Robert Gordon University Aberdeen UK; ^3^ Physical Activity for Health Research Centre University of Edinburgh Edinburgh UK; ^4^ Department of Health Services Research and Policy London School of Hygiene and Tropical Medicine London UK

**Keywords:** body composition, fat distribution, female, scoping review, subjective assessment

## Abstract

Body fat distribution is a key indicator of obesity‐related disease risk, often assessed through objective anthropometric measurements. However, objective implementation at scale is limited by measurement variability, cost, and anthropometrist skill. Subjective methods, widely applied in body image research, may offer an alternative but are less explored for determining obesity‐ and disease‐related risk. This scoping review aimed to identify the availability and characteristics of subjective body shape assessment tools for assessing regional body fat distribution in adult females. A search across five databases (inception to September 8, 2023), using terms for body shape and assessment tools, limited to females, yielded 13,646 unique records; 177 studies were included, reporting 80 tools (13 were variations of 7 originals). Studies utilized tools for varied purposes: body image/shape attractiveness, satisfaction, or distortion (73.4%); health/disease risk (18.1%); tool development/validation (13.0%); clothing/fashion (5.6%); or other (4.0%). Tools types included: figural (38.8%); photographic (21.3%); silhouette (16.3%); figural/scanned image with shape overlay (6.3%); computer generated image (6.3%); inanimate shape (3.8%); somatograph (1.3%); and unclassified (6.3%). Some tools were culturally adapted (e.g., modifying skin tone, clothing, or shape to the population), but most (17.6% of 51 applicable tools) depicted White ethnicity, limiting inclusivity. Among applicable tools, 56.3% included facial features, and 25.4% nakedness. This review reveals a variety of subjective tools, but limited application for disease‐related risk assessment. Further research should refine and culturally adapt subjective tools to ensure conceptual suitability, and validate their use for assessing obesity‐related disease risk.

**Abbreviations**
ABSI: A Body Shape Index
BMI: body mass index
BRI: Body Roundness Index
JND: just noticeable difference
PRISMA‐ScR: Preferred Reporting Items for Systematic Reviews and Meta‐Analyses extension for Scoping Reviews

## 1 Introduction

Obesity is commonly defined as excessive or abnormal body fat accumulation that impairs health [1]. According to the World Health Organization, more than 890 million adults worldwide are living with obesity [2], and in England, over one in four adults is classified as obese [3]. Obesity is strongly associated with adverse health outcomes, including type 2 diabetes, cardiovascular disease, certain cancers, and premature mortality [4, 6], making the identification of obesity a critical issue for both clinical and public health practice.

While body mass index (BMI) remains the accepted measurement for obesity [2], it is well known that BMI is a poor predictor of individual body fat or risk of cardiometabolic conditions, including type 2 diabetes mellitus, hypertension, and dyslipidaemia [7]. It is also recognized that centrally distributed body fat, particularly visceral fat, as postulated by Vague [8], is more closely related to the risk of pathologies such as coronary heart disease, stroke, and type II diabetes [9].

Objective anthropometric measurements such as waist and gluteal girth, as well as indices including waist–hip ratio, A Body Shape Index (ABSI) [10], and the Body Roundness Index (BRI) [11] have been used to estimate central adiposity, and studies have shown their usefulness for metabolic health risk prediction [12, 13, 14]. However, the implementation of such anthropometric measures at scale, or into routine clinical care, is not without challenge. Previous studies [15, 16, 17, 18, 19, 20, 21] exploring anthropometric protocols across a range of population groups highlight variability in measurements, independent of biological change, due to factors such as diurnal variation; accuracy and precision of the instruments; adherence to specific methods and procedures; the anthropometrist's technical capacity; and the methods of data recording. As a result, current gold standard protocols for anthropometric measurements [22] require training and expertise to ensure accuracy and reproducibility and can therefore be time‐consuming and costly to collect. Arguably, the level of accuracy required of measures to determine abdominal adiposity for general screening or population‐level studies may not need to be so high; the primary purpose is often to classify individuals into broad categories to identify trends, risk factors, and correlations, rather than to provide precise diagnoses. Self‐measures of body girths have been proposed as a solution to some of these practical limitations, and previous studies [23, 24] have provided some evidence on reliability, but can result in both over‐ and underestimation of adiposity. Improvements in the accuracy of self‐measures of waist girth have been shown using video instructions for participants, compared to written instructions [25]. However, the objective nature of the measurements still needs suitable equipment (e.g., a tape measure), adding to cost.

Subjective methods for assessing risk of obesity, such as photographs, silhouettes, and figure rating scales, are a low‐cost alternative to anthropometric techniques. Stunkard et al. [26] developed one of the earliest sets of silhouette showcards, presenting a series of nine sex‐specific body shapes, which has been used within research to explore body dissatisfaction. However, the drawn images in Stunkard's Figure Rating Scale (FRS) have been suggested to confound body shape with weight [27]. Additionally, previous authors have suggested bias in assessment may increase when using measurement scales that are not population specific [28], with Stunkard's scale being criticized for not reflecting the ethnic differences in body shape and distribution of fat [29]. Further, there is an absence of internationally agreed subjective measurements for use in research or routine clinical practice to assess obesity‐related risk.

Women experience distinct patterns of fat distribution and related metabolic risk compared to men [30], including a greater tendency toward subcutaneous fat accumulation pre‐menopause and a shift toward central adiposity post‐menopause [31]. Body image perception and the validity of visual rating scales have also been shown to differ by sex, with women generally displaying higher body dissatisfaction and greater sensitivity to body shape cues [32]. Therefore, this review focuses on women to reflect sex‐specific differences in fat distribution and psychosocial perceptions of body shape, which are important considerations when using subjective visual measures.

This scoping review aimed to determine the variety of simple visual subjective approaches to the evaluation of body fat distribution available, specifically with a focus on use in adult women.

## 2 Materials and Method

The study methods are reported according to the Preferred Reporting Items for Systematic Reviews and Meta‐analysis extension for scoping reviews (PRISMA‐ScR), and the study protocol was registered on Open Science Framework (https://doi.org/10.17605/OSF.IO/K2NQX).

### 2.1 Identifying the Research Question

Preliminary reviews of the literature on body shape assessment helped to refine the scope of the research protocol. This phase informed the decision to restrict the review to adult female populations, but to place no restrictions upon country because there are ethnic differences in female body shape and distribution of fat [33, 34].

The primary research questions were defined as follows: (1) Are simple visual subjective tools or rating scales to categorize female body shape or regional distribution of body fat available? (2) What are the principal characteristics of these instruments? (3) Do these subjective tools support categorization in terms of risk for adverse health, and have they been used to determine health risk?

For the purpose of this study, a visual subjective body shape assessment tool was defined as an item or resource that can be used by an individual (either self‐assessment or by an observer) to assess regional deposition of body fat that does not require any physical assessment. In this review, the term ‘body shape’ is used to refer to the external silhouette represented by subjective assessment tools. While these outlines do not directly measure body fat composition, they provide a practical proxy for underlying fat distribution (e.g., central vs. peripheral adiposity), which is relevant for disease risk.

### 2.2 Search Strategy

The search strategy was developed with input from a research librarian to ensure a comprehensive review of the available literature in the following databases: MEDLINE, EMBASE, CINAHL, Scopus, and Web of Science.

Relevant keywords for the search strategy were developed through test searches and piloting. Keywords or Medical Subject Headings (MeSH) terms relating to body shape (e.g., figure, physique, pear, gynoid, regional adiposity) and assessment tool (e.g., category, scale, rating), and limited to females, were developed. A tailored search strategy was developed for each database on the basis of identified key words (Table S1). No date limits were imposed, with searches completed in March 2022; updated September 8, 2023. Backward citation chaining was also conducted to identify additional relevant studies.

### 2.3 Selection of Eligible Studies

All search results were imported to Endnote 20.2 (Clarivate, Philadelphia, USA) for deduplication. Title and abstract screening were conducted independently by two authors, using the Rayyan web application for Systematic Reviews (https://www.rayyan.ai/) [35]. Full texts of potentially eligible studies retrieved were similarly screened independently by two authors (SCL screened all articles, and AH, GN, AB, and NH shared the duplicate screening) against the inclusion criteria. Screening discrepancies between reviewers were infrequent and were resolved through discussion; exact numbers were not recorded. Full text studies that did not meet the inclusion criteria were excluded.

### 2.4 Inclusion and Exclusion Criteria

This scoping review considered studies that: described the use of a visual subjective body shape assessment tool; the tool was presented in the paper, or the authors cited the original source so that the tool could be retrieved; the tool was designed for use with a female adult population (≥ 18 years). Studies were excluded if they used tools or scales that required objective measurement of body dimensions or composition for use (e.g., 3D body scanners) or if they described that a tool was used (e.g., silhouettes) without any means to view the tool directly. There was no limit on study design, language, or country of research/publication.

### 2.5 Data Charting and Synthesis

Data charting of the eligible studies was performed by one reviewer (SCL). Relevant data were charted using a pre‐specified and piloted form in Excel. Charted data included: first author, publication year, countries in which the included primary study was conducted, study aim, visual subjective body shape tool utilized, purpose of use, associations with health‐risk assessment, and details of objective measures used (excluding estimated measures) for comparison with the subjective tool. A 12% random sample of the charted data was validated by a second reviewer (AH), ensuring consistency, and there were no disagreements.

Tools utilized in the identified studies were coded in relation to visual tool type (figural, silhouette, photographic, etc.), categorized measurement scale type (nominal, ordinal, scale, mixed), modifications from original tools, race/ethnicity/skin tone where detailed or implied, the number of body shapes/categories, image color and orientation, and body/clothing detail represented and presence or absence of facial features. To ensure a transparent coding framework, the authors developed the following working definitions of visual tool types: figural (line or shaded body‐figure illustrations with some features), silhouette (outline/contour images with no internal detail), photographic (photo‐based images of real bodies), computer‐generated image (digitally modeled bodies rendered by software), inanimate shapes (abstract, non‐bodily forms), and somatograph (silhouette outlines derived from real photographs, depicting actual body contours). The data were analyzed descriptively, with tabulations used where appropriate to synthesize key findings in relation to the study objectives.

## 3 Results

**FIGURE 1:**
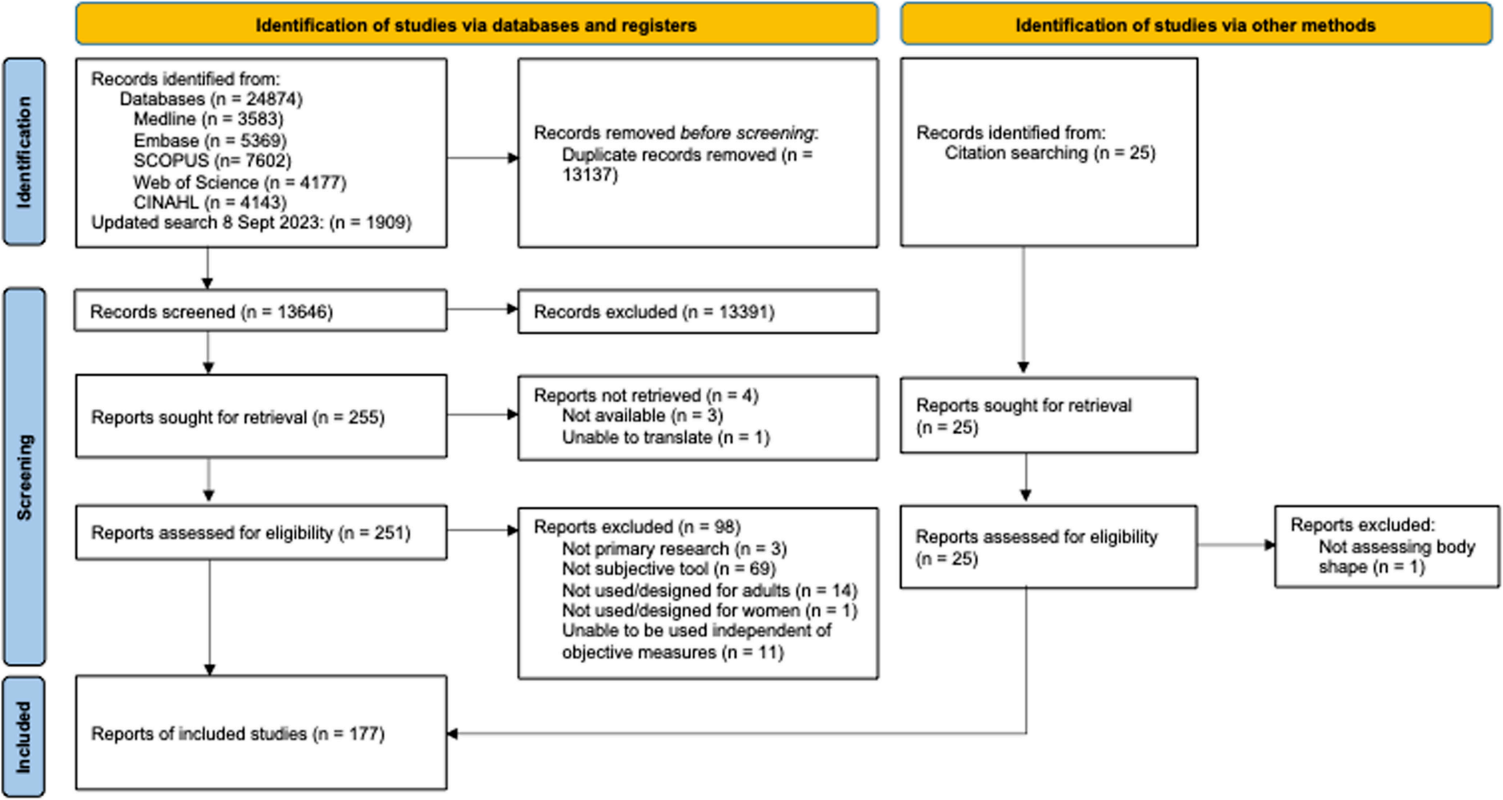
PRISMA flow‐chart [212] of the study selection process.

The database searches retrieved a total of 26,783 records, of which 13,137 were duplicates. A further 13,391 records were excluded after title and abstract screening. There were 255 potentially eligible studies taken forward for full text screening, and the full texts were retrieved for 251. One hundred and fifty‐three papers met all the eligibility criteria for inclusion in the scoping review, and an additional 24 studies were identified through hand searches of the reference lists, resulting in 177 included studies [27, 36, 37, 38, 39, 40, 41, 42, 43, 44, 45, 46, 47, 48, 49, 50, 51, 52, 53, 54, 55, 56, 57, 58, 59, 60, 61, 62, 63, 64, 65, 66, 67, 68, 69, 70, 71, 72, 73, 74, 75, 76, 77, 78, 79, 80, 81, 82, 83, 84, 85, 86, 87, 88, 89, 90, 91, 92, 93, 94, 95, 96, 97, 98, 99, 100, 101, 102, 103, 104, 105, 106, 107, 108, 109, 110, 111, 112, 113, 114, 115, 116, 117, 118, 119, 120, 121, 122, 123, 124, 125, 126, 127, 128, 129, 130, 131, 132, 133, 134, 135, 136, 137, 138, 139, 140, 141, 142, 143, 144, 145, 146, 147, 148, 149, 150, 151, 152, 153, 154, 155, 156, 157, 158, 159, 160, 161, 162, 163, 164, 165, 166, 167, 168, 169, 170, 171, 172, 173, 174, 175, 176, 177, 178, 179, 180, 181, 182, 183, 184, 185, 186, 187, 188, 189, 190, 191, 192, 193, 194, 195, 196, 197, 198, 199, 200, 201, 202, 203, 204, 205, 206, 207, 208, 209, 210, 211] (Table S2). Reasons for exclusion are reported in a PRISMA flow diagram [212] (Figure 1).

Most studies were from North America (42.4%, *n* = 75) or Europe (23.2%, *n* = 41) and published from 2000 onwards (72.9%, *n* = 129) (Table 1). The majority of studies utilized a subjective assessment tool to explore psychological aspects of body shape including body image, attractiveness, preferences, satisfaction, or distortion (73.4%, *n* = 130) with relatively fewer exploring perception or detection of health/disease risk (18.1%, *n* = 25). Table S3 shows the focus of the 32 studies where health/disease risk was explored, including where subjective assessments of participants were compared with objective measures. Eleven studies used the scales to determine obesity or metabolic health; 10 studies explored the relationship between body shape/size and specific diseases, predominantly breast cancer (*n* = 5) and type II diabetes mellitus (*n* = 3); and most others involved rating the perception of “healthiness” (*n* = 11). Of the 32 studies exploring health/disease risk, many (*n* = 15) did not correlate findings with objective measures. However, where objective measures were reported, BMI was most often used (*n* = 16).

**TABLE 1:** Characteristics and purposes of studies included in the review.

Variable	*n* (%)
Continent	
North America	75 (42.4%)
Europe	41 (23.2%)
Asia	20 (11.3%)
Africa	13 (7.3%)
South America	11 (6.2%)
Australia	9 (5.1%)
Dual	8 (4.5%)
Year of publication	
1970–1979	1 (0.6%)
1980–1989	10 (5.6%)
1990–1999	37 (20.9%)
2000–2009	54 (30.5%)
2010–2019	55 (31.1%)
2020–2023	20 (11.3%)
Purpose^a^	
Psychological aspects	130 (73.4%)
Health/disease	32 (18.1%)
Subjective tool development/validation	23 (13.0%)
Clothing/fashion	10 (5.6%)
Other^b^	7 (4.0%)

^a^ Cumulative percentage exceeds 100% as some studies had multiple purposes.

^b^ Other purposes such as factors influencing body shape judgments, physique stereotyping, influence of dietary pattern on body shape, risk factors for overweight and obesity, and exploring whether disliked body shapes transfer negative valency to foods.

Within included studies, 81 subjective body shape assessment tools were identified, with some studies utilizing more than one tool (Table S2/S4). Of these, 13 identified tools were variations of seven original tools, for example, where the original tool has been adapted to change the number of images, shapes, ethnicity, or features (Table S4). Sixty‐four studies (36%) utilized Stunkard's FRS [26] with a further six studies using a modified version of the FRS. Of all studies, 70.6% (*n* = 125) used a subjective assessment tool that was published prior to the year 2000 (Table S2), despite the majority of tools (55.5%, *n* = 45) being published since this time (Table S4). One tool [213] presented with insufficient details for the appraisal of characteristics, as it cited another source for the tool that was not possible to retrieve, and therefore was omitted from further analysis.

## 4 Characteristics of Subjective Body Shape Tools

An appraisal of all subjective tool characteristics is available in Table S4. Of 80 available tools, visual tool types were primarily figural (38.8%, *n* = 31) followed by photographic (21.3%, *n* = 17) or silhouette (16.3%, *n* = 13) (Table 2). The color of the tools was predominantly black and white (40.0%, *n* = 32), and orientation was front view (50.0%, *n* = 40). There were 76 tools where data were available on the number of images/shapes, and 49 utilized ≤ 9 representative body shapes (range 2–625, IQR 6–12.75).

**TABLE 2:** Characteristics of included visual assessment tools.

Variable	*n* (%)
Visual tool type	
Figural	31 (38.8%)
Photographic	17 (21.3%)
Silhouette	13 (16.3%)
Figural/scanned image with inanimate shape overlay	5 (6.3%)
Computer generated image	5 (6.3%)
Inanimate shapes	3 (3.8%)
Somatograph	1 (1.3%)
Unclassified	5 (6.3%)
Categorized measurement scale type	
Nominal	23 (28.8%)
Ordinal	40 (50.0%)
Scale	11 (13.8%)
Mixed	7 (8.8%)
Color	
Black and white	32 (40.0%)
Color	16 (20.0%)
Grayscale	7 (8.8%)
Not applicable (silhouette/shape) or not specified	25 (31.3%)
Orientation	
Front view	40 (50.0%)
Contrapposto ‘posed’ position	3 (3.8%)
Three‐quarter view	5 (6.3%)
Rear view	2 (2.5%)
Side view	1 (1.3%)
Multiple views	9 (11.3%)
Not applicable/specified	20 (25.0%)

Race/skin tone was explicit in 33.3% (*n* = 17) of 51 tools where an assessment could be made, with nine tools presenting White populations, six Black African, and two Asian (Bengali and Japanese). One study further amended the tool by Gruber et al. [214] by coloring the images a “light cool brown” color in order “to make them more credible for the three racial categories” (White, Hispanic, and African American/Black) examined in the study. One study created images specifically to be “racially neutral.” Nineteen (37.3%) tools presented figural‐based female shapes with an absence of shading.

Fifty‐three images presented the full body; eight tools presented a partial image of the body, with some features missing (*n* = 3 missing heads; *n* = 2 missing feet; *n* = 1 missing lower legs; *n* = 1 missing head and 2/3 of legs; *n* = 1 missing head, arms, and lower legs).

Excluding tools based on inanimate shapes and silhouettes/somatographs, 63 tools remained. Of those, 12.7% (*n* = 8) provided insufficient details to enable analysis of clothing represented. Swimwear was represented in 27.0% (*n* = 17) tools, 25.4% (*n* = 16) presented as naked, demonstrating definition of anatomical features such as the breasts, nipples, or genital area, 19.1% (*n* = 12) were fully clothed, and the remaining tools (17.5%, *n* = 11) presented the female bodies in underwear. Additionally, where an assessment of the presence of facial features could be made (*n* = 48 tools), facial features were apparent in 56.3% (*n* = 27) tools, although most were “stylized” to represent a face.

## 5 Discussion

This scoping review explored the research evidence pertaining to the subjective identification of female adult body fat distribution. Findings suggest a wide range of subjective body shape tools are available globally, with growing usage in recent decades. There was limited application of subjective tools for classifying health/disease risk. Instead, much of their use was within psychology‐based research relating to body shape preferences and attractiveness, or body image satisfaction or distortion, which may reflect growth in eating disorders over the last 50 years. [215] Where tools did have some health risk focus, they were largely applied to perceptions of ‘healthiness’ of a female body shape rather than obesity‐related risk. Additionally, studies demonstrate limited validation of health risk against objective measures of central adiposity such as WHR, more often utilizing measured BMI as a comparator, thus missing the potential for risk stratification according to body shape and body fat distribution. However, in a study of 131 women, Thoma et al. [199] demonstrated their subjective tool was a suitable proxy measure for the assessment of obesity and central adiposity, and Sangkum et al. [170] identified that the inclusion of subjective body shape improved the specificity of screening for obstructive sleep apnoea. With appropriate validation against accurate anthropometric measures, subjective tools for the assessment of body shape may be a low‐cost, reliable method for disease risk prediction and use in epidemiological studies.

### 5.1 Scale Size and Type

The majority of tools used an ordinal scale, but these present unique challenges in measurement due to the uneven intervals between categories. Gardner, Friedman, and Jackson [216] exemplified this issue using the most commonly used tool [26]; while the tool permits ranking of the body based on perceived attractiveness or other criteria, the distances between shapes/sizes are not consistent, with variation in proportional changes across chest and waist size between adjacent figures. This non‐uniformity poses difficulties in data interpretation; a one‐point shift on the scale may not necessarily indicate an equivalent change in body shape, undermining the reliability and validity of the measurements obtained.

The wide range of representative body shapes utilized within the identified tools prompts consideration of the practical implications of use. A higher number of body shapes may offer a more nuanced assessment of body shape perception, especially in diverse populations. Gardner et al. [216] highlight the importance of scale size representing what is considered a “just noticeable difference” (JND), defined as “the amount of change necessary in a stimulus for the change to be detected 50% of the time” [217]. However, because body shape changes in individuals may be spread across different body regions, it can be difficult to generate a scale representing all variations. Some authors have attempted diversification in this regard, for example, by presenting stimulus figures that represent different weight, waist girth, and hip girth categories [196]. However, those with a large number of choices [124, 125, 187] may introduce complexity that undermines their utility in large‐scale studies. Furthermore, it is possible that regardless of the scale size, participants may not use the full range of the scale, as they may not wish to be categorized at an extreme of body shape, which therefore leads to reducing their answers to a central tendency [216]. Striking a balance between comprehensiveness and simplicity is crucial when designing tools for assessing body shape perception in epidemiological research.

### 5.2 Image Orientation

In medical and scientific literature, the anatomical position is the reference position for most images, and in this scoping review, most tools utilized a single front‐facing view. Cornelissen et al. [66] conducted a study to determine whether the front view is optimal for the detection of body size changes and found that the three‐quarter view and side view stimuli performed better than the front view when exploring the JND for determining changes in body size using BMI. While this finding relates to body size rather than shape, Cohen et al. [60] found that in an African population, the correlation between abdominal obesity (waist circumference and waist‐hip ratio) and overall body shape was weaker from the front view compared to the side view. Alternative orientations were utilized in some studies, for example, the rear view by Kościński et al. [125], but the rationale was to “exclude the confounding effects of face and breast appearance on attractiveness perception” rather than to improve the prediction of body shape. Thus, image orientation is an important consideration, as it may affect both perception and accuracy when using visual tools to assess body shape.

### 5.3 Image Features

The details and features shown on visual body shape tools may have an influencing effect on the perception of body shape. Facial features may express emotions and may divert attention away from the evaluation of body shape. Noori et al. [218] identified that fearful and surprised facial features were perceived as slimmer compared to other facial features such as angry and neutral in a study of 70 men and women from a variety of ethnic groups. Talbot et al. [194] occluded the facial features in their computer‐generated images with the purpose of preventing participants from confusing facial attractiveness with body attractiveness. Additionally, facial features can vary significantly between cultural and ethnic groups; omitting them may therefore reduce distraction from facial cues and aid relatability across ethnicities. However, this approach addresses only one dimension of cultural variation.

Some tools identified in this review were adapted from the original to enhance cultural relevance to the population group under investigation. These adaptations included changes to skin shading, alterations to shape, and the addition of clothing. For example, Okoro and Oyejola [151] conducted a study on body image preferences of Nigerians with type 2 diabetes mellitus, utilizing Stunkard's FRS [26] but applying a “darker colour […] to represent the population of interest more accurately.” Nagasaka [143] modified the FRS to “more faithfully represent the shape of Japanese subjects,” and in the pilot stage of the research conducted by Greenhalgh, Chowdhury and Wood [104] “the naked figures were offensive to some Bangladeshis,” so an artist was asked to apply a watercolor wash representing traditional Bangladeshi clothing over the FRS images for use in the main study. However, in this scoping review, White females were depicted more often, and the prevalence of unshaded figural drawings implies a subtle visual cue that these body shape images should also be perceived as White. While it is essential to avoid reinforcing stereotypes, the known race/ethnic differences in body composition [33] must also be considered when developing or employing a visual subjective body shape tool. If a body shape tool is not aligned with the population's ethnicity, it may marginalize individuals whose body shapes diverge from the tool's predetermined standards, potentially fostering feelings of inadequacy and perpetuating societal biases. Inaccurate assessments due to cultural mismatch may also overlook health risks prevalent in particular ethnic groups, exacerbating existing health disparities. Recent empirical evidence underscores that this is not solely a matter of cultural relatability but also of perceptual accuracy. Ridley et al. [219] demonstrated that when participants were presented with body stimuli incongruent with their own ethnic identity, systematic misjudgments occurred, with East Asian and South Asian participants tending to overestimate, and White European participants to underestimate, body size by as much as three BMI units. These findings indicate that the use of ethnically mismatched stimuli can compromise both the validity of perceptual assessments and the cultural sensitivity of the tools employed. Furthermore, a lack of cultural sensitivity in a deployed tool can be perceived as disrespectful and undermine trust between researchers and communities. As societies become increasingly diverse, the need for culturally appropriate tools intensifies. However, navigating these challenges requires a delicate balance between cultural inclusivity and practical utility, underscoring the ongoing complexities in this field.

## 6 Strengths and Limitations

The key strength of this scoping review is the comprehensive, systematic searching of academic literature on subjective approaches to evaluating body fat distribution using the PRISMA‐ScR framework, following a publicly registered protocol prior to the commencement of the review. Furthermore, employing duplication of screening by reviewers enhanced the rigor and reliability of study selection, ensuring a more robust synthesis of evidence.

The chosen search terms were planned to encompass the breadth of research on the topic, but a potential limitation is the inclusion of studies which purport to relate to the assessment of body shape but instead conflate “size” with “shape.” As Garner, Jappe, and Gardner [220] highlight, many figural scales are based on artists' subjective depictions rather than verified body dimensions, resulting in distortions that may not correspond with the actual changes in body shape associated with weight gain or obesity. This issue raises questions about validity and suggests that some of the tools captured by the search may not have truly assessed body shape. Additionally, a small number of studies reported using a subjective tool, but the authors were unable to retrieve the source tool and therefore could not include these in the analysis.

## 7 Conclusion

This scoping review has highlighted that there are many subjective tools available for the identification of female body fat distribution, with variation in characteristics such as scale size and type, image orientation, features, and cultural representation. While these tools show promise in body image research, their application for health risk classification remains limited. Future research should therefore prioritize refining and culturally adapting these tools to ensure conceptual suitability and subsequently validate self‐identified body shape against accurate anthropometric measures to establish their reliability for scalable and low‐cost health/disease risk prediction and use in epidemiological studies and clinical practice.

## Author Contributions

S.C.L., N.H., L.V., and M.D.T. conceived the study and developed the research design and protocol. S.C.L. performed the literature searches and drafted the manuscript. S.C.L., A.H., G.N., A.B., and N.H. participated in abstract and title screening, as well as full text screening. S.C.L. conducted data extraction, with a sample checked by A.H. All authors critically reviewed drafts and edited the manuscript. All authors read and approved the final manuscript.

## Funding

The authors have nothing to report.

## Conflicts of Interest

The authors declare no conflicts of interest.

## Supporting information


**Table S1:** Search strategy.
**Table S2:** Included studies, aim, purpose, and identified body shape tool.
**Table S3:** Health risk and objective measures.
**Table S4:** Characteristics of body shape tools cited.

## Data Availability

Data sharing not applicable to this article as no datasets were generated or analysed during the current study.
