# Supplementary material for: Subjective Evaluation of Female Adult Body Fat Distribution: A Scoping Review
Source: Obes Rev. 2025 Dec 16;27(5):e70068. doi: 10.1111/obr.70068 (PMC13070896; doi:10.1111/obr.70068)
Supplement: Supplementary file 1 — Table S1: Search strategy. Table S2: Included studies, aim, purpose, and identified body shape tool. Table S3: Health risk and objective measures. Table S4: Characteristics of body shape tools cited. [file OBR-27-e70068-s001.docx]

TABLE S1. Search strategy

| MEDLINE AND EMBASE | |
| --- | --- |
| #1 | “body shape*” OR “body type*” OR “body figure” OR “physique” OR “android” OR “gynoid” OR “(apple or pear) adj5 shape* OR “(hourglass adj5 (shape or figure)” OR Body Fat Distribution/ OR “(regional adj4 fat) OR “regional adiposity” OR “(Adipose OR fat) adj4 tissue distribution)” |
| #2 | “categor*” OR “scale*” OR “rating” or “tool*” OR “assess*” OR “pictorial” OR “3D model” or “contour drawing” or “line drawing” OR “silhouette” OR “screening” OR “self-reported” |
| #3 | #1 AND #2 |
| #4 | Limit to human |
| #5 | Limit to female |

| SCOPUS | |
| --- | --- |
| #1 | “body shape*” OR “body type*” OR “body figure” OR physique OR android OR gynoid OR ((apple OR pear) W/5 shape*) OR (hourglass W/5 (shape OR figure)) OR “Body Fat Distribution“ OR (regional W/4 fat) OR “regional adiposity” OR ((Adipose OR fat) W/4 “tissue distribution”) |
| #2 | categor* OR scale* OR rating or tool* OR assess* OR pictorial OR “3D model” or “contour drawing” or “line drawing” OR silhouette OR screening OR “self-reported” |
| #3 | #1 AND #2 |
| #4 | female* OR Woman OR women |
| #5 | Limit to article or review |

| WEB OF SCIENCE | |
| --- | --- |
| #1 | “body shape*” OR “body type*” OR “body figure” OR physique OR android OR gynoid OR ((apple OR pear) NEAR/5 shape*) OR (hourglass NEAR/5 (shape OR figure)) OR “Body Fat Distribution“ OR (regional NEAR/4 fat) OR “regional adiposity” OR ((Adipose OR fat) NEAR/4 “tissue distribution”) |
| #2 | categor* OR scale* OR rating OR tool* OR assess* OR pictorial OR “3D model” or “contour drawing” or “line drawing” OR silhouette OR screening OR “self-reported” |
| #3 | #1 AND #2 |
| #4 | female* OR Woman OR women |
| #5 | Limit to article or review |

| CINAHL | |
| --- | --- |
| #1 | “body shape*” OR “body type*” OR “body figure” OR physique OR android OR gynoid OR “(apple OR pear) N5 shape*” OR “hourglass N5 (shape OR figure)” OR “Body Fat Distribution“ OR (regional N4 fat) OR “regional adiposity” OR ((Adipose OR fat) N4 “tissue distribution”) |
| #2 | “categor*” OR “scale*” OR “rating” or “tool*” OR “assess*” OR “pictorial” OR “3D model” or “contour drawing” or “line drawing” OR silhouette OR screening OR “self-reported” |
| #3 | #1 AND #2 |
| #4 | Limit to female |
| #5 | Limit to academic journals |

TABLE S2. Included studies, aim, purpose and identified body shape tool

| **Author (year)** | **Country** | **Study Aim** | **Purpose** | **Body Shape Tool used** |
| --- | --- | --- | --- | --- |
| Abdullah Ben-Ammar et al. (2013) ^46^ | Saudi Arabia | To 1) investigate the perception of body image among women in Hail province-SA, and 2) explore the differences in the motive and drive of body image and lifestyle attitude between married and single women. | Psychological aspects | Stunkard et al. (1983) ^26^ |
| Acevedo et al. (2014) ^36^ | Spain | To explore the knowledge of the weight, height, and body image perceptions of Spanish adults who were participating in a dietary consultation program. | Psychological aspects AND Health/ Disease | Stunkard et al. (1983) ^26^ |
| Albawardi et al. (2021) ^37^ | Saudi Arabia | To investigate the status of BI perception and to explore the associations of BD with demographics, PA, screen time, dietary habits and sleep among adult females attending fitness centers in Riyadh, the capital of Saudi Arabia. | Psychological aspects | Stunkard et al. (1983) ^26^ |
| Alexander et al. (2005) ^38^ | USA | To explore the relationships between body type and fit preferences with body cathexis, clothing benefits sought by consumers, and demographic profiles of consumers. | Clothing/ Fashion | Own tool |
| Aljadani (2019) ^39^ | Saudi Arabia | To 1) explore the correlation between BMI and BID, and 2) to investigate the prevalence of BID and body image perception across BMI categories among a sample (18-25 years old) of Young Saudi women in Makkah region. | Psychological aspects | Stunkard et al. (1983) ^26^ |
| Allison et al. (1993) ^40^ | USA | To test whether 1) there are ethnic’ differences in men’s preferences for degrees of fatness in women such that black and Hispanic men prefer fatter figures than do white men, and 2) there are ethnic differences in the range of women’s figures men will consider dating such that black and Hispanic men find a greater range of figures acceptable than do white men. | Psychological aspects | Williamson (1990) ^221^ |
| Amadou et al. (2014) ^41^ | Mexico | To explore the association between anthropometric factors, their modifications over lifetime, and breast cancer risk among Mexican women living in Mexico. | Health/ Disease | Tehard et al. (2002) ^198^ |
| Ard et al. (2007) ^42^ | USA | To determine 1) the cultural issues that affect the body weight of African American and White women who have a high body image disparity versus those that have a low or no body image disparity, and 2) the cultural issues that may influence a woman’s perception of her weight status. | Psychological aspects | Stunkard et al. (1983) ^26^ |
| Argnani et al. (2008) ^43^ | Italy | To examine the association between perceived body image and various demographic characteristics and Body Mass Index during several growth phases in a large representative sample from Emilia-Romagna region, northern Italy. | Psychological aspects | McElhone et al. (1999) ^222^ |
| Bays et al. (2009) ^44^ | USA | To examine the correlation between the Figure Rating Scale figures and BMI among individuals with and without diabetes mellitus. | Health/ Disease AND Subjective tool development/ validation | Stunkard et al. (1983) ^26^ |
| Beato-Fernández et al. (2009) ^45^ | Spain | To explore whether AN patients differ from BN patients and control participants with regard to the patterns of change in regional cerebral blood flow (rCBF) from baseline to their own body image exposure and from baseline to a neutral stimulus exposure? | Psychological aspects | Gardner et al. (1999) ^98^ |
| Bentley et al. (2005) ^47^ | Malawi | To 1) better understand breast-feeding practices in Lilongwe, especially the perceived ability of HIV-positive women to breast-feed their infants exclusively for 6 mo, 2) to explore the perceived relationship between maternal health and exclusive breast-feeding, and 3) explore the perceived association between illness and body image. | Psychological aspects AND Health/ Disease | Own tool |
| Bhuiyan et al. (2003) ^48^ | USA | To assess differences in body image perception among race-gender groups in the community. | Psychological aspects | Stunkard et al. (1983) ^26^ |
| Bizuneh et al. (2023) ^49^ | Ethiopia | To assess the body characteristics (body size, garment sizes worn, shape and body satisfaction) of Ethiopian young adult female consumers and their effects on garment fit satisfaction and fit preferences of ready-to-wear garments such as T-shirts, blouses/shirts, skirts and jeans trousers. | Psychological aspects AND Clothing/ Fashion | Simmons et al. (2004) ^180^ |
| Bjerggaard et al. (2015) ^50^ | Denmark | To 1) determine whether the relationship between self-reported body image and BMI differed across BMI categories among men and women without known diabetes, and 2) determine whether self-reported body image was associated with screen-detected type 2 diabetes or pre-diabetes after adjustment for objectively measured obesity. | Health/ Disease | Stunkard et al. (1983) ^26^ |
| Boukrim et al. (2022) ^51^ | Morocco | To identify the main risk factors associated with overweight and obesity among women in southern Morocco. | Risk factors for overweight and obesity | Stunkard et al. (1983) ^26^ |
| Braun et al. (2006) ^52^ | USA | To determine the extent to which the desirability of a potential mate is affected by body shape (waist-to-shoulder ratio or waist-to-hip ratio) and/or agreeableness. | Psychological aspects | Own tool |
| Brodie et al. (1996) ^53^ | UK | To establish people's preconceived notions of body image and examine such preconceptions in relation to current physical activity | Psychological aspects | Furnham & Ahbhai (1983) ^91^ |
| Brown et al. (2014) ^54^ | USA | To explore, describe and understand this population’s personal view of body [as measured by a body image scale] related to 1) reality (the actual/current body image), 2) ideal body image, and 3) healthy body image. To determine gender differences and associations between the perception of these three categories of body images as assessed by responses to the Pulvers Body Image Scale. | Psychological aspects | Pulvers et al. (2004) ^161^ |
| Butler et al. (1993) ^55^ | USA | To explore physique-stereotyping using participant generated traits. | Physique stereotyping | Ryckman et al. (1989) ^223^ |
| Capers et al. (2016) ^56^ | USA | To illustrate why body shape assessments may be useful in clinical practice above and beyond BMI, and its relationship to health risk among a sample of AA and EA women by 1) pictorially presenting different body shapes at the same BMI among pre- and postmenopausal women pairs by race/ethnicity, and 2) examining associations between body shape and health outcomes after controlling for BMI and menopausal status within racial/ethnic groups. | Health/ Disease | Own tool |
| Cohen et al. (2001) ^58^ | USA | To investigate the role of three physical variables (waist-to-hip ratio, breast size, and body weight) in attraction among lesbian and bisexual women. | Psychological aspects | Singh & Young (1995) ^186^ |
| Cohen et al. (2011) ^59^ | Cameroon | To explore body weight perceptions in Cameroon by developing and validating a human photographic stimuli and a body image assessment guide (BIAG) taking into account the biocultural characteristics of the population. | Health/ Disease AND Subjective tool development/ validation | Own tool |
| Cohen et al. (2015) ^60^ | Africa | To develop and validate the Body Size Scale (BSS): a new scale of photos of real models, more representative of African phenotypes with regard to body size, and taking into account more measures of adiposity. | Health/ Disease AND Subjective tool development/ validation | Own tool |
| Cohen et al. (2020) ^61^ | Africa | To develop and validate the BOSHAS to evaluate body shape perceptions related body image disorders in African populations. | Psychological aspects | Own tool |
| Cohn et al. (1992) ^62^ | USA | To examine female and male perceptions of ideal body figures for themselves, same sex peers and other sex peers. | Psychological aspects | Stunkard et al. (1983) ^26^ |
| Collins et al. (1988) ^63^ | Australia | To 1) examine the accuracy of body recognition during adolescence, and 2) replicate previous findings: differential accuracy of recognition of body image between males and females, somatotypic preference, and the relationships among objective, subjective, and ideal body images. | Psychological aspects | Own tool |
| Connolly et al. (2004) ^64^ | Australia | To chart changes in preferences for specific male and female body shapes from early childhood to adulthood. | Psychological aspects | Singh (1993) ^181^ |
| Cornelissen et al. (2009) ^65^ | UK | To determine the eye-movements made when male and female observers rated a set of female images for attractiveness, body fat and WHR. | Psychological aspects | Own tool |
| Cornelissen et al. (2018) ^66^ | UK | To determine which of three stimulus orientations: frontal, three-quarter or side view, is most suitable for use in body size estimation tasks. | Subjective tool development/ validation | Own tool |
| Costa et al. (2019) ^67^ | Brazil | To verify how middle-aged individuals age and elderly people perceive their body image. | Psychological aspects | Stunkard et al. (1983) ^26^ |
| Da Silva-Filho et al. (2008) ^68^ | Brazil | To investigate the risk factors derived from the morphological and functional measures of anthropometry, associated with the body image of the male and female genders in individuals who practice walking in the city of Natal, Brazil. | Psychological aspects | Stunkard et al. (1983) ^26^ |
| Da Silva et al. (2016) ^69^ | Brazil | To investigate body composition, dietetic profile, self-perceived body image and social desirability among professional ballet dancers. | Psychological aspects | Stunkard et al. (1983) ^26^ |
| Davis (1985) ^70^ | USA | To 1) investigate the relationship between perceived somatotype and body-cathexis for female subjects, 2) determine the perceived ideal somatotype of college females, and 3) examine the relationship between perceived somatotype and attitudes toward fashion and clothing use. | Psychological aspects AND Clothing/ Fashion | Own tool |
| Davis (1990) ^71^ | Canada | To determine whether avid female exercisers have a greater degree of body narcissism and place greater emphasis on their appearance than do non-exercising women. | Psychological aspects | Own tool |
| De Lauzon-Guillain et al. (2010) ^72^ | France | To examine the influence of birth weight and body silhouette, from childhood to middle age, on incident diabetes in a cohort of middle-aged French women born between 1925 and 1950. | Health/ Disease | Stunkard et al. (1983) ^26^ |
| De Medeiros et al. (2016) ^73^ | Brazil | To evaluate the somatotype and body image of people living with HIV/AIDS. | Psychological aspects | Stunkard et al. (1983) ^26^ |
| Deeks et al. (2001) ^74^ | Australia | To explore women‘s evaluation of appearance, health, fitness, preoccupation with weight, overall body satisfaction, and visual perception of body image. | Psychological aspects | Stunkard et al. (1983) ^26^ |
| Demarest et al. (2000) ^75^ | USA | To examine gender, ethnic, and age differences in body-shape dissatisfaction and in the amount of distortion in estimating the attractiveness preferences of the opposite sex. | Psychological aspects | Stunkard et al. (1983) ^26^ |
| Douty et al. (1984) ^76^ | USA | To 1) devise a method for studying the attractiveness attribution process that would be more objective, informative, and systematic than the usual global response method that is frequently used, 2) create a more realistic situation by using representative stimuli of real people, with their multiple characteristics, to replace abstracted drawings of figures and isolated variables that are sometimes used, and 3) obtain assessment responses of young male and female subjects to these figures of young women to identify the figure characteristics that influence their judgements. | Psychological aspects | Douty 1968^224^; Douty & Brannon (1984) ^76^ |
| Dratva et al. (2016) ^77^ | Iceland, Norway, Sweden, Denmark and Estonia | To investigate the predictive power of figural scales to identify individuals at metabolic risk. | Subjective tool development/ validation AND Health/ Disease | Own tool |
| Duda et al. (2007) ^78^ | Ghana | To determine 1) if Ghanaian women preferred to be of a larger body size, 2) if women were aware of obesity-linked illnesses, and 3) if there was sufficient motivation to reduce weight by conventional means of diet and exercise for a healthier life. | Psychological aspects AND Health/ Disease | Own tool |
| Duncan et al. (2005) ^79^ | UK | To examine the effect of random presentation of figures on individuals’ figure ratings. | Subjective tool development/ validation | Stunkard et al. (1983) ^26^ |
| Ejike (2015) ^80^ | Nigeria | To investigate the prevalence of weight misperception, weight preference, and BSD among Nigerians and to assess the impact of these factors on population quality of life (QOL). | Psychological aspects | Stunkard et al. (1983) ^26^ |
| Epstein et al. (1991) ^81^ | USA | To assess the relative benefits of methods for estimating percent overweight within families, as well as the potential for using matching of silhouettes for diagnosing obesity. | Health/ Disease AND Subjective tool development/ validation | Stunkard et al. (1983) ^26^ |
| Fagherazzi et al. (2013) ^82^ | France | To examine the association between the risk for breast cancer in adulthood and both body shape at different ages (age 8, menarche, ages 20–25, and 35–40 years) and body shape trajectories throughout life using group-based trajectory modeling, taking into account menopausal status and tumor hormone receptor status. | Health/ Disease | Stunkard et al. (1983) ^26^ |
| Fagherazzi et al. (2015) ^83^ | France | To examine influence of the evolution of body shape over the lifespan on T2D risk in adulthood. | Health/ Disease | Stunkard et al. (1983) ^26^ |
| Fallon et al. (1985) ^84^ | USA | To 1) compare current and ideal figures with ratings of most attractive figures by the subject and by members of the opposite sex, and 2) examine both dissatisfaction with current appearance and distortions in estimating the attractiveness preferences of the opposite sex. | Psychological aspects | Stunkard et al. (1983) ^26^ |
| Ferrer-Garcia et al. (2008) ^85^ | Spain | To analyze the psychometric characteristics of the Body Image Assessment Software (BIAS), an innovative interactive computer program developed to assess body image disturbances. | Psychological aspects | Beebe, Holmbeck and Grzeskiewicz (1999) ^225^ |
| Ford et al. (1990) ^86^ | Egypt | To explore whether women who are exposed to Western views, yet living within their own culture, appraise their bodies in similar ways to Western women. | Psychological aspects | Stunkard et al. (1983) ^26^ |
| Forestell et al. (1998) ^87^ | Canada | To extend Tassinary and Hansen’s (1998) findings by providing a more detailed analysis of how individual characteristics of body weight, waist and hip size independently and interactively influence women’s perceptions of attractiveness. | Psychological aspects | Tassinary and Hansen 1998^196^ |
| Forestell et al. (2004) ^27^ | Canada | To determine whether female undergraduate restrainers differed from non-restrainers in how attractive they perceived female line drawings. | Psychological aspects | Tassinary and Hansen 1998^196^ |
| Foroni et al. (2011) ^88^ | USA | To examine the effects of a label’s semantic content on the strength of categorization effects. | Factors influencing body shape judgements | Own tool adapted from Furnham and Alibhai (1983) ^91^ |
| Foroni et al. (2013) ^89^ | USA | To explore whether a category label system, once established, produces long-term effects. | Factors influencing body shape judgements | Furnham and Alibhai (1983) ^91^ |
| Franko et al. (2013) ^90^ | USA | To 1) examine trends over time in cover images in Latina magazine, and 2) to investigate the frequency of body modification and body acceptance articles in this publication. | Psychological aspects | Pulvers et al. (2004) ^161^ |
| Furnham et al. (1983) ^91^ | UK | To investigate the perception of body shapes by females from different cultures but similar socio-economic backgrounds. | Psychological aspects | Own tool |
| Furnham et al. (1994) ^92^ | UK and Uganda | To compare body attitude ratings of fairly large groups of British and Ugandan males and females using established male and female figure drawings. | Psychological aspects | Furnham and Alibhai (1983) ^91^ |
| Furnham et al. (1997) ^94^ | UK and Singapore | To investigate cross-cultural differences in the perception of male and female body shapes as a function of exercise. | Psychological aspects AND Health/ Disease | Furnham and Alibhai (1983) ^91^ |
| Furnham et al. (1990) ^93^ | UK | To investigate the area of "normal figures" in an attempt to quantify and qualify the specific somatic preferences of males and females, using systematically manipulated anatomical drawings of a female body. | Psychological aspects | Own tool |
| Furnham et al. (1994) ^97^ | UK | To investigate the perception of body shapes by females from four different exercising backgrounds but similar socio-economic backgrounds | Psychological aspects | Own tool |
| Furnham et al. (1997) ^96^ | UK | To investigate the perception and meta-perception of male and female body shapes, differing along two dimensions: weight and waist-to-hip ratio. | Psychological aspects | Singh (1993) ^181^ |
| Furnham et al. (2002) ^95^ | UK, Greece and Uganda | To investigate cross-culturally the effect of WHR and weight on attractiveness. | Psychological aspects | Own tool but based on Tassinary and Hansen 1998^196^ |
| Gardner et al. (1994) ^99^ | USA | To investigate the feasibility of using a TV-video methodology to investigate the influence of somatotype on personality evaluation utilizing video images of live models. | Psychological aspects | Own tool |
| Gardner et al. (1999) ^98^ | USA | To develop and validate two new and improved tools for assessment of body-image. | Subjective tool development/ validation | Own tool |
| Gilbert-Diamond et al. (2009) ^100^ | Colombia | To examine 1) the associations of socioeconomic factors with overweight and obesity based on BMI among adult women in Bogota, Colombia, and 2) the associations between socioeconomic factors and the women’s identification of the Stunkard silhouettes that most closely resembled their current body shape, ideal body shape, and healthiest perceived body shape. | Psychological aspects | Stunkard et al. (1983) ^26^ |
| Goldberg et al. (1996) ^101^ | USA | To develop a new visual image rating scale for females with realistic three-dimensional contour and different types of fat and muscle distribution. | Psychological aspects AND Subjective tool development/ validation | Own tool |
| Grant et al. (2015) ^102^ | Australia | To assess if there was an association between midlife parental body shape and four measures of obesity and fat distribution among Australian adults. | Health/ Disease | Stunkard et al. (1983) ^26^ |
| Greenberg et al. (1996) ^103^ | USA | To examine racial differences in body type preferences, and how these preferences might be translated into real-life behaviors. | Psychological aspects | Own tool adapted from Stunkard et al. (1983) ^26^ |
| Greenhalgh et al. (2005) ^104^ | UK | To explore body image perception and its relation to health in British Bangladeshis with diabetes | Psychological aspects AND Health/ Disease | Own tool adapted from Stunkard et al. (1983) ^26^ |
| Guy et al. (1980) ^105^ | USA | To explore the relationship between sex role stereotyping and body types. | Psychological aspects | Own tool |
| Hallinan (1988) ^106^ | Australia | To explore Muslim and Judaic-Christian mens' perceptions of desirable female body shape. | Psychological aspects | Stunkard et al. (1983) ^26^ |
| Hallinan et al. (1991) ^107^ | USA | To examine collegiate athletes' and nonathletes' perceptions of their current and desired body images by testing both men and women. | Psychological aspects | Stunkard et al. (1983) ^26^ |
| Hallinan et al. (1993) ^108^ | USA | To 1) assess the association of regular physical exercise on the body-shape perceptions of elderly women, and 2) examine (within the elderly study group) the association of age with body perception. | Psychological aspects | Stunkard et al. (1983) ^26^ |
| Han et al. (1999) ^109^ | UK | To explore whether overweight and central fat distribution make people look older and have poorer health. | Health/ Disease | Own tool |
| Hasan et al. (2022) ^110^ | UAE | To explore the effect of fat free mass index (FFM-I) on the agreement between perceived and actual body image, shape and BMI among a convenient sample of university students in the United Arab Emirates (UAE). | Psychological aspects | Stunkard et al. (1983) ^26^ |
| Horvath (1981) ^111^ | Canada | To explore the influence of selected torso parameters on defining human physical attractiveness. | Psychological aspects | Own tool |
| Hunter et al. (2021) ^112^ | USA | To develop a valid measure of body image related to the curvy ideal. | Psychological aspects | Own tool |
| Hussain et al. (2010) ^113^ | Norway | To investigate body size perceptions among Pakistani women enrolled in a lifestyle intervention in Oslo. | Psychological aspects | Stunkard et al. (1983) ^26^ |
| Izydorczyk (2013) ^114^ | Poland | To examine selected psychological traits and body image characteristics in a population of young Polish females suffering from BED. | Psychological aspects | Thompson & Gray (1995) |
| Jackson et al. (2014) ^115^ | USA | To examine the association between body image and depressive symptoms in a sample of midlife women in generally good health, using a multidimensional perspective of body image. | Psychological aspects | Stunkard et al. (1983) ^26^ |
| Jansen et al. (2022) ^116^ | Germany | To investigate 1) the explicit and implicit affective attitudes toward under-, normal- and overweight women in a sample of young women, and 2) the relation to body satisfaction and the contributing factors. | Psychological aspects | Swami et al. (2008) ^226^ |
| Kakeshita et al. (2009) ^117^ | Brazil | To 1) develop silhouette scales adapted to the Brazilian socio-economic and cultural context, for adults and children, and 2) test their reliability as an initial step in their validation and standardization process. | Subjective tool development/ validation | Own tool |
| Kamaria et al. (2016) ^118^ | Malaysia | To explore the relationship of BSD with body shape concern and body image perception among young adults who are studying in a university. | Psychological aspects | Nagasaka et al. (2008) ^143^ |
| Kapoor et al. (2022) ^119^ | India | To 1) estimate the prevalence of BID, its pattern and the factors associated with it among female undergraduate students, and 2) assess the level of concern about their body image. | Psychological aspects | Thompson & Gray (1995) ^201^ |
| Kapoor et al. (2022) ^120^ | India | To 1) assess the eating behavior and level of self‐esteem among undergraduate female college students, 2) explore the relationship of these with body image perception, and 3) identify the factors associated with high‐risk eating behavior and low self‐esteem. | Psychological aspects | Thompson & Gray (1995) ^201^ |
| Kaufer-Horwitz et al. (2006) ^121^ | Mexico | To establish the correlation between perceived body size (through self-selected silhouettes) and measured BMI in Mexican adult men and women and to determine the BMI values that correspond to each silhouette. | Health/ Disease AND Subjective tool development/ validation | Stunkard et al. (1983) ^26^ |
| Kirkpatrick et al. (1978) ^122^ | USA | To identify whether temperament judgements associated with different body types differ according to sex and age of the respondents? | Psychological aspects | Sheldon (1940) ^227^ |
| Kościcka et al. (2016) ^123^ | Poland | To 1) assess body attitudes and body perception among children and their parents, 2) to analyse the predictors of attitudes towards the body in study groups, and 3) to evaluate mediation between the variables: body dissatisfaction, media pressure and children’s ideal silhouette. | Psychological aspects | Thompson & Gray (1995) ^201^ |
| Kościński (2013) ^124^ | Poland | To determine the most preferred BMI and WHR values and the relative importance of these traits for attractiveness of women’s bodies by using stimuli free of the known weaknesses. | Psychological aspects | Own tool |
| Kościński (2014) ^125^ | Poland | To determine the most preferred female WHR by using silhouette stimuli free of previously established weaknesses. | Psychological aspects | Own tool |
| Lascelles et al. (2003) ^126^ | UK | To investigate whether conditioned shifts in the affective valences of foodstuffs can be affected through their pairing with particular types of body shapes. | Exploring whether disliked body shapes transfer negative valency to foods | Own tool |
| Leães et al. (2019) ^127^ | Brazil | To determine the anthropometric profile and health indicators, including QoL, of patients with pituitary adenomas before and after pituitary surgery | Psychological aspects | Stunkard et al. (1983) ^26^ |
| Lee et al. (2020) ^128^ | Malaysia | To determine the gender differences in body image perception and its association with body mass index and dietary intake among university students. | Psychological aspects | Nagasaka et al. (2008) ^143^ |
| Lenart et al. (1995) ^129^ | USA | To investigate the relationship between physical activity and body-image preference | Psychological aspects AND Subjective tool development/ validation | Own tool developed from Goldberg (1996) ^101^ |
| Liao et al. (2022) ^130^ | China | To examine the reliability and validity of recalled body shape as well as the discriminative degree of obesity classification via recalled body shape among the Chinese rural population. | Health/ Disease AND Subjective tool development/ validation | Stunkard et al. (1983) ^26^ |
| Liburd et al. (1999) ^131^ | USA | To explore perceptions of black women with diabetes about their own bodies, their ideas about body size and shape, and their opinions on the personal and environmental factors that influence their preferences about body size and shape. | Psychological aspects AND Health/ Disease | Own tool |
| Lôbo et al. 2020) ^132^ | Brazil | To evaluate the subjective perception of body image and satisfaction with body shapes between men and women | Psychological aspects | Kakeshita et al. (2009) ^117^ |
| Mahmud et al. (2007) ^133^ | Australia and Pakistan | To compare body image attitudes in Muslim Pakistani and non-Muslim Caucasian Australian young women | Psychological aspects | Stunkard et al. (1983) ^26^ |
| Manuel et al. (2010) ^134^ | USA | To identify any statistically significant differences in body cathexis and clothing benefits sought between African-American women who have similar body shapes and fit preferences. | Clothing/ Fashion | Own tool |
| Markey et al. (2004) ^135^ | USA | To determine 1) women’s body satisfaction, 2) their beliefs about how satisfied their husbands are with their bodies, and 3) how satisfied their husbands actually are with their bodies. | Psychological aspects | Stunkard et al. (1983) ^26^ |
| Mciza et al. (2005) ^136^ | South Africa | To develop (adapting existing instruments to suit the multi-ethnic community of South African women) and validate (determine their cultural sensitivity) instruments for assessing body image constructs in South African women and preadolescent schoolgirls. | Subjective tool development/ validation | Stunkard et al. (1983) ^26^ |
| Mintem et al. (2015) ^137^ | Brazil | To identify how changes in BMI from childhood to adulthood influenced body image satisfaction at 23 years of age in subjects from a cohort study who have been followed-up since birth. | Psychological aspects | Stunkard et al. (1983) ^26^ |
| Mo et al. (2014) ^138^ | China, Hong Kong and UK | To explore the relative importance of socioeconomic status and the 'visual diet mechanism' in determining body preferences. | Psychological aspects | Tovée et al. (1999) ^203^ |
| Mooney et al. (1994) ^139^ | USA | To investigate whether impressions of a female target would vary as a function of her preference for a high fat or low fat diet. | Psychological aspects | Stunkard et al. (1983) ^26^ |
| Murnen et al. (2015) ^140^ | USA | To examine the body ideals that heterosexual college women and men chose for romantic partners to determine the extent to which they reflect unrealistic ideals promoted in the mass media. | Psychological aspects | Tantleff-Dunn & Thompson (2000) ^213^; Thompson & Gray (1995) ^201^; Singh (1993) ^181^ |
| Musaiger et al. (2004) ^141^ | Qatar | To determine the relationship between social factors, as well as body mass index, with body-shape preferences for men and women among Arab women living in Qatar. | Psychological aspects | Stunkard et al. (1983) ^26^ |
| Musher-Eizenman et al. (2003) ^142^ | USA | To examine the relationship between parental weight and related attitudes about body size (their own and their child’s) and feeding practices for their children. | Psychological aspects | Own tool |
| Nagasaka et al. (2008) ^143^ | Japan | To evaluate the correlation between self-rating body size and BMI derived from measured height and weight in adult Japanese men and women. | Health/ Disease AND Subjective tool development/ validation | Own tool adapted from Stunkard et al. (1983) ^26^ |
| Naigaga et al. (2018) ^144^ | Algeria | To describe body size perceptions and preferences in different age groups among adult Saharawi refugees | Psychological aspects | Stunkard et al. (1983) ^26^ |
| Naor-Ziv et al. (2020) ^145^ | Israel | To 1) investigate the hierarchical structure of the body image using a rank-ordering task of specifically constructed body shapes, and 2) present two analytical procedures that, when used together, enable us to portray this structure in a 2D representational space | Psychological aspects | Swami et al. (2008) ^226^ |
| Németh et al. (2021) ^146^ | Hungary | To examine the associations of women between the ages of 20 and 65 with lingerie advertising, and how the perception of lingerie models relates to the Women with satisfaction with their own bodies. | Psychological aspects | Thompson & Gray (1995) ^201^ |
| Newcomb et al. (2011) ^147^ | USA | To 1) explore Hispanic women’s fit preferences for casual tops, pants, skirts, and dresses, and 2) understand the characteristics that shape these preferences by analyzing whether self-reported physical body characteristics of body shape perception, BMI, and clothing size, affected apparel fit preferences. | Clothing/ Fashion | Simmons et al. (2004) ^180^ |
| Nichols et al. (2009) ^148^ | Trinidad and Tobago | To determine the behavioural correlates of body dissatisfaction among students at the University of the West Indies, St Augustine campus, Trinidad and Tobago. | Psychological aspects | Own tool |
| Nikishina et al. (2018) ^149^ | Russia | To study impairments to body scheme in patients with meningioma of the parietal-occipital area before and after surgical treatment. | Psychological aspects | Own tool |
| Novella et al. (2015) ^150^ | USA | To 1) provide psychometric support for a realistic continua of bodies that include substantial extremes in terms of obesity and muscularity for both men and women, termed the Presentation of Images on a Continuum Scale (PICS) and, 2) to show a consistent pattern of perceptual discrepancies between current, ideal, and attractive body selections on the PICS. | Psychological aspects AND Subjective tool development/ validation | Own tool |
| Okoro et al. (2008) ^151^ | Nigeria | To explore the perception of current and preferred body figures of individuals with type 2 diabetes and/or hypertension, and their preferred body figure for the opposite gender. | Psychological aspects | Becker et al. (1999) ^228^ adapted from Stunkard et al. (1983) ^26^ |
| Pandarum et al. (2017) ^152^ | South Africa | To provide the South African apparel industry with a better perspective on the sizing and fit concerns currently encountered by South African consumers, and a greater insight into why these occur. | Clothing/ Fashion | Own tool adapted from Liddlelow (2011) What’ your body shape? at  https://www.style- makeover-hq.com/body-shape.html |
| Parent et al. (1996) ^153^ | Canada | To explore the assessment of familial obesity patterns, using a subjective measure, for determining the role of obesity in breast cancer development. | Health/ Disease | Stunkard et al. (1983) ^26^ |
| Pawlowski et al. (2008) ^154^ | Poland | To test whether the perception of female body shape, and breast and face attractiveness by males changes in relation to season. | Psychological aspects | Rozmus-Wrzesinska & Pawlowski (2005) ^167^ |
| Pazhoohi et al. (2020) ^155^ | Portugal | To explore the behavioral and neurophysiological correlates of processing female body figures with different breast sizes and WHRs, providing insights into the cognitive mechanisms responsive to the processing of these bodily features and their influence on attractiveness. | Psychological aspects | Own tool |
| Pisut et al. (2007) ^156^ | USA | To investigate fit preferences of female consumers in the USA based on the relationship between fit preferences, body cathexis, fit problems, and personal profiles. | Clothing/ Fashion | Own tool |
| Portnoy (1993) ^157^ | USA | To explore 1) to what extent the elderly are influenced by body type attractiveness perceptions, 2) whether age or sex are systematically related to older persons' attraction preferences, and 3) how an older person's self-identified body type influences body-type attractiveness preferences of ageing peers. | Psychological aspects | Own tool |
| Prasad et al. (2015) ^158^ | India | To measure the prevalence and pattern of Body shape Dissatisfaction among polytechnic college students in Puducherry. | Psychological aspects | Stunkard et al. (1983) ^26^ |
| Price et al. (2014) ^159^ | Canada | To describe the body shape expectations and self-ideal body shape discrepancy using the SFRS in women seeking LSG surgery in Newfoundland and Labrador (NL), Canada. | Psychological aspects | Stunkard et al. (1983) ^26^ |
| Puja et al. (2020) ^160^ | India | To determine the proportion of college girls who overestimate/ underestimate their body image measured using Figure Rating Scale (FRS) as compared to their Body Mass Index (BMI) and to assess the influence of media, peers and parents on their perception of body image. | Psychological aspects | Stunkard et al. (1983) ^26^ |
| Pulvers et al. (2004) ^161^ | USA | To 1) develop an instrument in which the figures were detailed and human-like but not specific to any one culture, and 2) evaluate the psychometric properties and cultural relevance of the scale among African Americans. | Subjective tool development/ validation | Own tool |
| Ribeiro et al. (2013) ^162^ | Brazil | To evaluate the different perceptions of body size and shape (normal, real and ideal) of patients who were preoperatively bariatric surgery compared to patients coming from different postoperatively time. | Psychological aspects | Stunkard et al. (1983) ^26^ |
| Romieu et al. (2012) ^163^ | Mexico | To evaluate the association of self-perceived body shape silhouette and BMI with dietary patterns. | Influence of dietary pattern on body shape | Tehard et al. (2002) ^198^ |
| Rosen et al. (1993) ^164^ | USA | To explore the preferences for a side-view silhouette of a female figure in African-American and white college males. | Psychological aspects | Own tool adapted from Bell et al. (1986) ^229^ |
| Roy et al. (2006) ^165^ | USA | To examine the relationship between physiological (aerobic fitness, participation in physical activity, and percent body fat) and socioeconomic variables with body-shape perception and dissatisfaction among college-age students attending an all women's institution. | Psychological aspects | Stunkard et al. (1983) ^26^ |
| Rozin et al. (2001) ^166^ | USA | To explore the stability of body image and body image dissatisfaction over a 15 year period using a single subjective tool. | Psychological aspects | Stunkard et al. (1983) ^26^ |
| Rozmus-Wrzesinska et al. (2005) ^167^ | Poland | To test whether in westernized society men’s perception of female body shape attractiveness is influenced more by hip or by waist size (particularly, in relation to the diverse biological signals they carry). | Psychological aspects | Own tool |
| Safir et al. (2005) ^168^ | Israel | To examine influences of gender and cultural background on participants’ satisfaction with body-shape. | Psychological aspects | Stunkard et al. (1983) ^26^ |
| Sands et al. (2004) ^169^ | Australia | To investigate the validity of a method for assessing body image satisfaction based on computer manipulation of a digitized image of self. | Psychological aspects | Stunkard et al. (1983) ^26^ |
| Sangkum et al. (2017) ^170^ | USA | To investigate the effect of using the presence (or absence) of an apple body type, in addition to BMI, on diagnostic performance of the STOP-BANG questionnaire for obstructive sleep apnoea. | Health/ Disease | Images from Mayo Clinic, used with permission |
| Santana et al. (2019) ^171^ | Brazil | To compare the body image and body esthetic between two groups of women with different levels of physical activity. | Psychological aspects | Kakeshita et al. (2009) ^117^ |
| Santo André et al. (2022) ^172^ | Brazil | To explore experiences, perceptions, and feelings regarding eating attitudes associated with EDs symptoms and body image (dis)satisfaction in classical ballet dancers using a mixed-method approach. | Psychological aspects | Stunkard et al. (1983) ^26^ |
| Sarabia-Cobo (2012) ^57^ | Spain | To study the concept of body image in elderly people and its association with the prevalence of depressive and anxiety disorders. | Psychological aspects | Stunkard et al. (1983) ^26^ |
| Saucedo-Molina et al. (2017) ^173^ | Mexico and Canada | To 1) examine and compare disordered eating behaviors, body thin-ideal internalization (BTHIN), and body image dissatisfaction (BID) in Mexican and Canadian university women, and 2) identify and describe its relationship with body mass index (BMI) and waist circumference (WC). | Psychological aspects | Gomez -Peresmitré et al. (2000) ^230^ |
| Schützwohl (2006) ^174^ | Germany | To test a new methodological approach to male attractiveness judgments of female waist-to-hip ratio. | Psychological aspects AND Health/ Disease | Singh (1993) ^181^ |
| Seo et al. (2018) ^175^ | USA | To explore actual ready-to-wear clothing fit for African American female college students and their satisfaction with it. | Clothing/ Fashion | Own tool |
| Šerifović et al. (2005) ^176^ | Bosnia and USA | To investigate the relationship between body dissatisfaction and stress in university women from Bosnia and from United States and to compare body dissatisfaction as well as judgments of their personal and cultural ideal body shape. | Psychological aspects | Stunkard et al. (1983) ^26^ |
| Serpa et al. (2017) ^177^ | Portugal | To analyze and understand relationships existing between the level of physical activity, the composition body, perception and satisfaction with body image in university students from the Algarve (Portugal). | Psychological aspects | Stunkard et al. (1983) ^26^ |
| Shelton et al. (2011) ^178^ | USA | To compare the self-reported body shape (gynoid vs android) with dual energy x-ray absorptiometry (DXA) acquired lumbar spine and hip neck BMD measurements in peri-menopausal African-American women. | Health/ Disease | Own tool |
| Shih et al. (2022) ^179^ | Taiwan | To investigate gender differences in body figure ratings as to how students perceive their body shape and the degree of body satisfaction of undergraduate students in Hualien, eastern Taiwan. | Psychological aspects | Collins (1991) ^231^ |
| Simmons et al. (2004) ^180^ | USA | To 1) determine if the current sizing systems meet the needs of today’s female population, and 2) develop preliminary subgroups for the female population that might aid in the description of their various shapes. | Clothing/ Fashion | Own tool |
| Singh (1993) ^181^ | USA | To 1) explore the role played by WHR in female attractiveness, and 2) ascertain whether men perceive WHR to be correlated with some component of female fitness. | Psychological aspects | Own tool |
| Singh (2004) ^185^ | Azore Islands and Guinea-Bissau | To explore cultural differences in attractiveness judgements and stereotypes. | Psychological aspects | Singh (1993) ^181^ |
| Singh (1994) ^183^ | USA | To investigate the relation between body fat distribution, as measured by waist-to-hip ratio (WHR), and perception of desirable female body shape in college-age black men and women. | Psychological aspects | Singh (1993) ^181^ |
| Singh (1994) ^182^ | USA | To investigate the interrelationships of female body fat distribution as measured by the waist-to-hip ratio (WHR), overall body size, perceived attractiveness, youthfulness, health, and need to lose weight. | Psychological aspects AND Health/ Disease | Own tool |
| Singh (1994) ^184^ | USA | To invstigate 1) whether physicians would correctly infer reproductive and health states of a woman on the basis of the size of WHR, and 2) to determine whether physicians would also associate attractiveness and other personal attributes with thinness. | Psychological aspects AND Health/ Disease | Singh (1993) ^181^ |
| Singh et al. (1995) ^186^ | USA | To determine how variations in overall body fat, body fat distribution, as measured by waist-to-hip ratio, breast size, and hip width interact and affect the judgment of female age, attractiveness, and desirability for romantic relationships. | Psychological aspects | Own tool |
| Smith et al. (2007) ^187^ | UK | To explore what makes the shape of a woman’s body attractive. | Psychological aspects | Own tool |
| Stevens et al. (1998) ^188^ | USA | To investigate body figure ratings in a much more broadly based sample and across a much wider age range of Australian women (not college or university students). | Psychological aspects | Stunkard et al. (1983) ^26^ |
| Strauman et al. (1994) ^189^ | USA | To investigate the relation between self-concept and body-image disturbance in selected female undergraduates. | Psychological aspects | Williamson et al. (1989) ^232^ |
| Streeter et al. (2003) ^190^ | USA | To evaluate attractiveness judgements on a range of waist, hip, and chest sizes. | Psychological aspects | Own tool |
| Suzuki (2007) ^191^ | Japan | To evaluate a new body silhouette scale. | Subjective tool development/ validation | Own tool |
| Tabande et al. (2012) ^192^ | Iran | To measure the body image of patients with breast cancer in Golestan province, Northeast of Iran. | Health/ Disease | Stunkard et al. (1983) ^26^ |
| Talbot et al. (2022) ^193^ | Australia | To 1) examine self-ratings of *actual* and *ideal* bodies and the attractiveness rating of the body of another gender using two bi-dimensional figural rating scales, and 2) explore if there are associations between body preferences and self-body ratings in men and women for both muscularity and body fat. | Psychological aspects | Talbot et al. (2023) ^194^ |
| Talbot et al. (2023) ^194^ | Australia | To examine 1) content, convergent, concurrent, and discriminant validity of the SM‐F utilizing measures of body dissatisfaction, eating disorder symptoms, body mass index (BMI), and depression, anxiety, and stress, and 2) the specific validity evidence for the SM‐F relating to muscularity and body fat. | Subjective tool development/ validation | Own tool |
| Taren et al. (1999) ^195^ | USA | To determine how specific psychological characteristics would be associated with under-reporting and measured TEE using doubly labelled water as a reference method. | Psychological aspects | Stunkard et al. (1983) ^26^ |
| Tassinary et al. (1998) ^196^ | USA | To test the waist:hip ratio hypothesis of female physical attractiveness by independently varying weight, waist and hip size. | Psychological aspects AND Subjective tool development/ validation | Own tool |
| Tehard et al. (2002) ^198^ | France | To 1) assess the validity of self-reported measurements of anthropometric characteristics and body silhouette, 2) test the relationship between the body silhouettes and the BMI derived from the self-measured weight and height, and 3) analyze factors related to misclassification of the self-reported body silhouettes. | Subjective tool development/ validation | 8 images from Stunkard et al. (1983) ^26^ |
| Tehard et al. (2005) ^197^ | France | To examine the relation between breast cancer and body shape at adolescence. | Health/ Disease | Stunkard et al. (1983) ^26^ |
| Thoma et al. (2012) ^199^ | USA | To assess whether reproductive-aged women adequately perceive and report their shape for incorporation into women’s health and life course studies. | Health/ Disease | Own tool; Stunkard et al. (1983) ^26^ |
| Thompson et al. (1995) ^201^ | USA | To create a set of detailed, front-view contour drawings of each sex, for the assessment of body image, without the limitations of figures previously in use. | Subjective tool development/ validation | Own tool |
| Thompson et al. (1988) ^200^ | USA | To replicate and extend of Fallon and Rozin’s (1985) investigation of body image. | Psychological aspects | Stunkard et al. (1983) ^26^ |
| Tovée et al. (2002) ^202^ | UK | To determine whether shape, in the form of WHR, can play a role in determining attractiveness. | Psychological aspects | Own tool |
| Tovée et al. (1999) ^203^ | UK | To 1) investigate the relative importance of BMI and WHR in the perception of female attractiveness, 2) explore what visual cues can be used to give an accurate and reliable measure of an individual's BMI, and 3) explore differences with previous studies. | Psychological aspects | Own tool |
| Tutkuviene et al. (2018) ^204^ | Lithuania | To assess women's self-esteem, body image and weight control before and during pregnancy. | Psychological aspects | Stunkard et al. (1983) ^26^ |
| Velov et al. (2023) ^205^ | Serbia | To determine the attitude of respondents towards the importance attached to the physical appearance of the female body in the modern socio-cultural context and the degree of (dis)satisfaction with its own body. | Psychological aspects | Swami et al. (2008) ^226^ |
| Vuruskan et al. (2011) ^206^ | Turkey | To explore female body shape classification groups and to look for an objective method to classify the body shapes based on body dimensions. | Clothing/ Fashion | Own tool |
| Wetsman et al. (1999) ^207^ | Tanzania and USA | To explore the WHR and weight preferences among Hadza foragers. | Psychological aspects AND Health/ Disease | Singh (1993) ^181^ |
| Yanover et al. (2010) ^208^ | USA | To 1) investigate how individuals categorize the weight status of other individuals, 2) to evaluate whether muscularity level moderated ratings, and 3) whether two figures with equal adiposity but different levels of muscularity receive equivalent weight status ratings. | Factors influencing body shape judgements | Gruber et al. (1999) ^214^ |
| Yates et al. (2004) ^209^ | USA | To distinguish body mass index (BMI) and body/ self-dissatisfaction among Asian subgroups. | Psychological aspects | Furnham and Alibhai (1983) ^91^ |
| Zellner et al. (1989) ^210^ | USA | To explore the effects of eating abnormalities and gender on perceptions of desirable body shape. | Psychological aspects | Stunkard et al. (1983) ^26^ |
| Ziegler et al. (2005) ^211^ | USA | To: 1) assess the energy, macro and micro-nutrient intakes of female US international synchronized skaters; and 2) assess the body image perceptions and weight-related attitudes of female US international synchronized skaters. | Psychological aspects | Stunkard et al. (1983) ^26^ |

TABLE S3. Health Risk and Objective measures

| **Author (Year)** | **Health risk explored** | **Was subjective assessment compared with objective measures?** |
| --- | --- | --- |
| Acevedo et al. (2014) ^36^ | Perceptions of 'healthiness' | Yes. BMI |
| Amadou et al. (2014) ^41^ | Breast cancer | Yes. BMI |
| Bays et al. (2009) ^44^ | Obesity | Yes. Self-measured WC |
| Bentley et al. (2005) ^47^ | Perceptions of 'healthiness' | No |
| Bjerggaard et al. (2015) ^50^ | Type II Diabetes Mellitus | Yes. BMI |
| Capers et al. (2016) ^56^ | Metabolic risk | Yes. BMI, total body fat (%), android-gynoid ratio, trunk fat (kg), Leg fat (kg) |
| Cohen et al. (2011) ^59^ | Obesity | Yes. BMI |
| Cohen et al. (2015) ^60^ | Overweight and obesity | Yes. Height, weight, BMI, WC, HC, WHR, % body fat, biepicondylar humerus, biepicondylar femur, endomorphy, mesopmorphy, ectomorphy |
| De Lauzon-Guillain et al. (2010) ^72^ | Type II Diabetes Mellitus | No |
| Dratva et al. (2016) ^77^ | Metabolic risk | Yes. BMI, WC |
| Duda et al. (2007) ^78^ | Perceptions of 'healthiness' | Yes. BMI |
| Epstein et al. (1991) ^81^ | Obesity | No. |
| Fagherazzi et al. (2013) ^82^ | Breast cancer | No |
| Fagherazzi et al. (2015) ^83^ | Type II Diabetes Mellitus | No |
| Furnham et al. (1997) ^94^ | Perceptions of 'healthiness' | No |
| Grant et al. (2015) ^102^ | Obesity | No |
| Greenhalgh et al. (2005) ^104^ | Perceptions of 'healthiness' | Yes. BMI |
| Han et al. (1999) ^109^ | Perceptions of 'healthiness' | Yes. BMI, WHR |
| Kaufer-Horwitz et al. (2006) ^121^ | Overweight and obesity | Yes. Height, weight, BMI |
| Liao et al. (2022) ^130^ | Obesity | Yes. Height, weight, BMI, WC |
| Liburd et al. (1999) ^131^ | Perceptions of 'healthiness' | No |
| Nagasaka et al. (2008) ^143^ | Obesity | Yes. Height, weight, BMI |
| Parent et al. (1996) ^153^ | Breast cancer | No |
| Sangkum et al. (2017) ^170^ | Obstructive Sleep Apnoea | Yes. BMI, WHR, Neck circumference |
| Schützwohl (2006) ^174^ | Perceptions of 'healthiness' | No |
| Shelton et al. (2011) ^178^ | Osteoporosis | Yes. Height, weight, BMI, Hip neck BMD, Lumbar spine BMD |
| Singh (1994) ^182^ | Perceptions of 'healthiness' | No |
| Singh (1994) ^184^ | Perceptions of 'healthiness' | No |
| Tabande et al. (2012) ^192^ | Breast cancer | No |
| Tehard et al. (2005) ^197^ | Breast cancer | No |
| Thoma et al. (2012) ^199^ | Obesity and central adiposity. | Yes. Height, weight, BMI, circumferences (chest, hip, waist), CHR, CWR, WHR, WHtR, arm fat index (AFI), centripetal fat ratio (CFR) |
| Wetsman et al. (1999) ^207^ | Perceptions of 'healthiness' | No |

TABLE S4: Characteristics of body shape tools cited

| **Author (year)** | **Categorised measurement scale type** | **Modified from** | **Race/ Ethnicity/ Skintone** | **Colour** | **Full or partial image** | **No. shapes/ categories** | **Visual tool type** | **Orientation of image** | **Clothing** | **Facial features** |
| --- | --- | --- | --- | --- | --- | --- | --- | --- | --- | --- |
| Alexander et al. (2005) ^38^ | Nominal | n/a | Not stated | n/a | n/a | 4 | Shape | n/a | n/a | n/a |
| Becker et al. (1999) ^228^ | Ordinal | Stunkard et al. (1983) ^26^ (altered skintone) | Black | C | Full | 9 | Figural | Front | Swimwear | Yes |
| Beebe et al. (1999) ^225^ | Ordinal | n/a | n/a | n/a | Not stated (image not provided) | 9 | Silhouette | n/a | n/a | n/a |
| Bell et al. (1986) ^229^ | Ordinal | n/a | n/a | n/a | Full | 8 | Silhouette | n/a | n/a | n/a |
| Bentley et al. (2005) ^47^ | Ordinal | Stunkard et al. (1983) ^26^ (altered skintone) | Black | G | Full | 9 | Figural | Front | Underwear | Yes |
| Braun et al. (2006) ^52^ | Ordinal | n/a | Not stated | B&W | Not stated | 2 | Photographic | Not stated | Not stated | Not stated |
| Capers et al. (2016) ^56^ | Ordinal | n/a | n/a | n/a | Full | 16 | Silhouette | Front, Side | n/a | n/a |
| Cohen et al. (2011) ^59^ | Nominal | n/a | Black | B&W | Full | 6 | Photographic | Front | Fully clothed | Occluded |
| Cohen et al. (2015) ^60^ | Ordinal | n/a | Black | C | Full | 9 | Photographic | Front, Side | Fully clothed | Occluded |
| Cohen et al. (2020) ^61^ | Nominal | n/a | Black | C | Full | 10 | Photographic | Front, Side | Fully clothed | Occluded |
| Collins et al. (1988) ^63^ | Ordinal | n/a | No | B&W | Full | 19 | Figural | Front, Side, Rear | Naked | No |
| Collins et al. (1991) ^231^ | Ordinal | n/a | Figural image without shading | B&W | Full | 7 | Figural | Front | Swimwear | Yes |
| Cornelissen et al. (2009) ^65^ | Ordinal | n/a | White | C | Full | 46 | Photographic | Front | Underwear | Occluded |
| Cornelissen et al. (2018) ^66^ | Ordinal | n/a | White | B&W | Full | 13 | CGI | Front, Side, Three-quarter | Underwear | Yes |
| Davis (1985) ^70^ | Ordinal | n/a | Figural image without shading | B&W | Full | 7 | Figural | Front | Naked | No |
| Davis (1990) ^71^ | Ordinal | n/a | Not stated | B&W | Not stated | 5 | Figural | Not stated | Swimwear | Not stated |
| Douty (1968) ^224^ | Nominal | n/a | n/a | n/a | Full | 5 | Silhouette | Front | n/a | n/a |
| Douty et al. (1984) ^76^ | Ordinal | n/a | n/a | B&W | Partial (missing feet) | 29 | Somatograph | Side, Posterior | n/a | n/a |
| Dratva et al. (2016) ^77^ | Nominal | Stunkard et al. (1983) ^26^ (colour image) | White | C | Full | 9 | Figural | Front | Swimwear | Yes |
| Duda et al. (2007) ^78^ | Nominal | n/a | Black | G | Full | 12 | Figural | Front | Swimwear | Yes |
| Foroni et al. (2011) ^88^ | Nominal | Furnham & Alibhai (1983) ^91^ | Figural image without shading | B&W | Full | 9 | Figural | Front | Fully clothed | No |
| Furnham et al. (1983) ^91^ | Ordinal | n/a | Figural image without shading | B&W | Full | 12 | Figural | Front | Naked | No |
| Furnham et al. (1990) ^93^ | Nominal | n/a | Figural image without shading | B&W | Full | 12 | Figural | Front | Naked | No |
| Furnham et al. (1994) ^97^ | Ordinal | n/a | Figural image without shading | B&W | Full | 9 | Figural | Front | Naked | No |
| Furnham et al. (2002) ^95^ | Ordinal | Tassinary & Hansen 1998^196^ | Not stated | B&W | Full | 8 | Figural | Three-quarter | Swimwear | No |
| Gardner et al. (1994) ^99^ | Ordinal | n/a | Not stated | C | Full | 3 | Photographic | Front | Fully clothed | Yes |
| Gardner et al. (1999) ^98^ | Scale | n/a | n/a | n/a | Full | 13 | Silhouette | Front | n/a | n/a |
| Goldberg et al. (1996) ^101^ | Scale | n/a | White | G | Full | 15 | Photographic | Front | Naked | Yes |
| Gómez-Peresmitré et al. (2000) ^230^ | Ordinal | n/a | Not stated | Not stated | Not stated | 9 | Not stated | Not stated | Not stated | Not stated |
| Greenberg et al. (1996) ^103^ | Ordinal | n/a | “racially neutral” | B&W | Full | 9 | Figural | Front | Swimwear | Yes |
| Greenhalgh et al. (2005) ^104^ | Ordinal | Stunkard et al. (1983) ^26^ (Bengali dress watercolour wash over body) | Asian (Bangladeshi) | B&W | Full | 9 | Figural | Front | Fully clothed | Yes |
| Gruber et al. (1999) ^214^ | Ordinal | n/a | “light cool brown” | C | Full | 9 | Figural | Front | Underwear | No |
| Guy et al. (1980) ^105^ | Nominal | n/a | n/a | n/a | - | 3 | Silhouette | n/a | n/a | n/a |
| Han et al. (1999) ^109^ | Ordinal | n/a | n/a | n/a | Full | 8 | Silhouette | Front, Side | n/a | n/a |
| Horvath (1979) ^233^ | Scale | n/a | Figural image without shading | B&W | Full | 11 | Figural | Front | Naked | No |
| Hunter et al. (2021) ^112^ | Scale | n/a | No | G | Full | 25 | CGI | Front | Naked | Yes |
| Kakeshita et al. (2009) ^117^ | Ordinal | n/a | Figural image without shading | B&W | Full | 15 | Figural | Front | Swimwear | No |
| Kościński (2013) ^124^ | Scale | n/a | White | C | Full | 312 | Photographic | Rear | Underwear | n/a |
| Kościński (2014) ^125^ | Ordinal | Kościński (2013) ^124^ (Fewer categories) | White | C | Full | 156 | Photographic | Rear | Underwear | n/a |
| Lascelles et al. (2003) ^126^ | Ordinal | n/a | Not stated | C | Full | 15 | Photographic | Not stated | Naked and “semi-naked” | Occluded |
| Lenart et al. (1995) ^129^ | Scale | Goldberg et al. (1996) ^101^ (used selected images, then generated new versions with accentuated muscularity and/or truncal padding) | White | G | Full | 30 | Photographic | Front | Naked | Yes |
| Liburd et al. (1999) ^131^ | Ordinal | n/a | n/a | B&W | Full | 3 | Silhouette | n/a | n/a | n/a |
| Manuel et al. (2010) ^134^ | Nominal | n/a | Figural image without shading | B&W | Full | 4 | Figural/ scanned image with shape overlay | Front | Naked | Yes |
| McElhone et al. (1999) ^222^ | Nominal | n/a | Not stated | Not stated | Not stated | 9 | Not stated | Not stated | Not stated | Not stated |
| Musher-Eizenman et al. (2003) ^142^ | Ordinal | n/a | Not stated | Not stated | Not stated | Not stated | Not stated | Not stated | Not stated | Yes |
| Nagasaka et al. (2008) ^143^ | Ordinal | Stunkard et al. (1983) ^26^ (features altered, and thinner silhouettes, for Japanese audience) | Asian (Japanese) | B&W | Full | 9 | Figural | Front | Swimwear | Yes |
| Nichols et al. (2009) ^148^ | Ordinal | n/a | n/a | n/a | Not stated | 7 | Silhouette | n/a | n/a | n/a |
| Nikishina et al. (2018) ^149^ | Nominal | n/a | n/a | n/a | Not stated | 3 | Silhouette | n/a | n/a | n/a |
| Novella et al. (2015) ^150^ | Ordinal | n/a | No | C | Partial (missing head) | 16 | Figural | Front | Underwear | n/a |
| Pandarum et al. (2017) ^152^ | Nominal | n/a | Figural image without shading | B&W | Full | 6 | Figural/ scanned image with shape overlay | Front | Swimwear | Yes |
| Pazhoohi et al. (2020) ^155^ | Mixed | n/a | No | B&W | Partial (missing head and 2/3 of legs) | 6 | CGI | Front | Swimwear | n/a |
| Pisut et al. (2007) ^156^ | Nominal | n/a | Figural image without shading | B&W | Full | 4 | Figural/ scanned image with shape overlay | Front | Naked | Yes |
| Portnoy (1993) ^157^ | Nominal | n/a | n/a | n/a | Not stated | 3 | Silhouette | n/a | n/a | n/a |
| Pulvers et al. (2004) ^161^ | Nominal | n/a | No | B&W | Full | 9 | Figural | Front | Underwear | Yes |
| Rosen et al. (1993) ^164^ | Ordinal | n/a | Not stated | Not stated | Not stated | Not stated | Not stated | Side | Not stated | Not stated |
| Rozmus-Wrzesinska et al. (2005) ^167^ | Scale | n/a | White | B&W | Partial (missing feet) | 10 | Photographic | Front, Rear | Full clothed | Yes |
| Ryckman et al. (1989) ^223^ | Mixed | n/a | Not stated | Not stated | Not stated | 3 | Figural | Not stated | Not stated | Not stated |
| Sangkum et al. (2017) ^170^ | Nominal | n/a | No | C | Partial (missing lower legs) | 2 | Figural/ scanned image with shape overlay | Front | Naked | No |
| Seo et al. (2018) ^175^ | Nominal | n/a | Not stated | Not stated | Not stated | 4 | Not stated | Not stated | Not stated | Not stated |
| Sheldon (1940) ^227^ | Nominal | n/a | Figural image without shading | B&W | Full | 9 | Figural | Front, Side | Naked | Yes |
| Shelton (2011) ^178^ | Nominal | n/a | Not stated | Not stated | Not stated | Not stated | Not stated | Not stated | Not stated | Not stated |
| Simmons et al. (2004) ^180^ | Nominal | n/a | Not stated | n/a | n/a | 7 | Shape | n/a | n/a | n/a |
| Singh (1993) ^181^ | Mixed | n/a | Figural image without shading | B&W | Full | 12 | Figural | Contrapposto | Swimwear | Yes |
| Singh (1994) ^182^ | Mixed | Singh (1993) ^181^  (Fewer categories) | Figural image without shading | B&W | Full | 6 | Figural | Contrapposto | Swimwear | Yes |
| Singh et al. (1995) ^186^ | Mixed | Singh (1993) ^181^  (fewer categories and adapted for large and small breasts) | Figural image without shading | B&W | Full | 8 | Figural | Contrapposto | Swimwear | Yes |
| Smith et al. (2007) ^187^ | Scale | n/a | No | G | Partial (missing head, arms, and lower legs) | 625 | Photographic | Front | Fully clothed | n/a |
| Streeter et al. (2003) ^190^ | Mixed | n/a | Not stated | Not stated | Not stated | 27 | Photographic | Three-quarter | Fully Clothed | Not stated |
| Stunkard et al. (1983) ^26^ | Ordinal | n/a | Figural image without shading | B&W | Full | 9 | Figural | Front | Swimwear | Yes |
| Suzuki (2007) ^191^ | Scale | n/a | No | B&W | Full | 9 | CGI | Three-quarter | Naked | Yes |
| Swami et al. (2008) ^226^ | Scale | Tovee et al. (1999) ^203^ (selected images) | No | G | Partial (missing head) | 10 | Photographic | Front | Fully clothed | n/a |
| Talbot et al. (2023) ^194^ | Scale | n/a | White | C | Full | 34 | CGI | Front | Underwear | Occluded |
| Tassinary et al. (1998) ^196^ | Mixed | n/a | Figural image without shading | B&W | Full | 27 | Figural | Three-quarter | Swimwear | No |
| Tehard et al. (2002) ^198^ | Ordinal | Stunkard et al. (1983) ^26^ (only 8 shapes/ categories) | Figural image without shading | B&W | Full | 8 | Figural | Front | Swimwear | Yes |
| Thoma et al. (2012) ^199^ | Nominal | n/a | n/a | n/a | n/a | 4 | Shape | n/a | n/a | n/a |
| Thompson et al. (1995) ^201^ | Ordinal | n/a | Figural image without shading | B&W | Full | 9 | Figural | Front | Underwear | Yes |
| Tovée et al. (1999) ^203^ | Ordinal | n/a | No | C | Full | 50 | Photographic | Front | Fully clothed | Occluded |
| Tovée et al. (2002) ^202^ | Ordinal | n/a | No | C | Partial (missing head) | 60 | Photographic | Front | Fully clothed | n/a |
| Vuruskan et al. (2011) ^206^ | Nominal | n/a | No | C | Full | 5 | Figural/ scanned image with shape overlay | Front | Underwear | No |
| Williamson et al. (1989) ^232^ | Ordinal | n/a | n/a | n/a | Not stated | 9 | Silhouette | Not stated | n/a | n/a |
| Williamson et al. (1990) ^221^ | Ordinal | n/a | No | B&W | Full | 9 | Figural | Three-quarter | Naked | No |

B&W = Black & White, G = Grayscale, C = Colour, n/a = not applicable, not stated = no image available with insufficient written description

**References (not otherwise cited in main document)**

[221] D. A. Williamson, C. J. Davis, E. G. Duchmann, S. J. McKenzie, and P. C. Watkins, *Assessment of Eating Disorders: Obesity, Anorexia, and Bulimia Nervosa* (Pergamon Press, 1990).

[222] S. McElhone, J. M. Kearney, I. Giachetti, H.‐J. F. Zunft, and J. A. Martínez, “Body Image Perception in Relation to Recent Weight Changes and Strategies for Weight Loss in a Nationally Representative Sample in the European Union,” *Public Health Nutrition* 2, no. 1a (1999): 143–151.

[223] R. M. Ryckman, M. A. Robbins, L. M. Kaczor, and J. A. Gold, “Male and Female Raters' Stereotyping of Male and Female Physiques,” *Personality and Social Psychology Bulletin*15, no. 2 (1989): 244–251.

[224] H. I. Douty, “Visual Somatometry in Health Related Research,” *Journal of the Alabama Academy of Science*39, no. 1 (1968): 21–24.

[225] D. W. Beebe, G. N. Holmbeck, and C. Grzeskiewicz, “Normative and Psychometric Data on the Body Image Assessment‐Revised,” *Journal of Personality Assessment* 73, no. 3 (1999): 374–394.

[226] V. Swami, N. Salem, A. Furnham, and M. J. Tovée, “Initial Examination of the Validity and Reliability of the Female Photographic Figure Rating Scale for Body Image Assessment,” *Personality and Individual Differences*44, no. 8 (2008): 1752–1761.

[227] W. H. Sheldon, S. S. Stevens, and W. B. Tucker, “The Varieties of Human Physique,” (1940).

[228] D. M. Becker, L. R. Yanek, D. M. Koffman, and Y. C. Bronner, “Body Image Preferences Among Urban African Americans and Whites From Low Income Communities,” *Ethnicity & Disease* 9, no. 3 (1999): 377–386.

[229] C. Bell, S. W. Kirkpatrick, and R. C. Rinn, “Body Image of Anorexic, Obese, and Normal Females,” *Journal of Clinical Psychology*42, no. 3 (1986): 431–439.

[230] G. Gómez‐Peresmitré, A. Granados, J. Jáuregui, G. Pineda Garcia, and S. A. Tafoya Ramos, “Un Instrumentopara Medir Imagen Corporal: Versión Computarizada y de Papel y Lápiz,” *Revista Mexicana de Psicología* (2000): 1-9.

[231] M. E. Collins, “Body Figure Perceptions and Preferences Among Preadolescent Children,” *International Journal of Eating Disorders* 10, no. 2 (1991): 199–208.

[232] D. A. Williamson, C. J. Davis, A. J. Goreczny, and D. C. Blouin, “Body‐Image Disturbances in Bulimia Nervosa: Influences of Actual Body Size,” *Journal of Abnormal Psychology* 98, no. 1 (1989): 97–99.

[233] T. Horvath, “Correlates of Physical Beauty in Men and Women,” *Social Behaviors and Personality* 7, no. 2 (1979): 145–150.
